# Orlistat Mitigates Oxidative Stress-Linked Myocardial Damage via NF-κβ- and Caspase-Dependent Activities in Obese Rats

**DOI:** 10.3390/ijms231810266

**Published:** 2022-09-06

**Authors:** Zaidatul Akmal Othman, Zaida Zakaria, Joseph Bagi Suleiman, Khairul Mohd Fadzli Mustaffa, Nur Asyilla Che Jalil, Wan Syaheedah Wan Ghazali, Ninie Nadia Zulkipli, Mahaneem Mohamed

**Affiliations:** 1 Unit of Physiology, Faculty of Medicine, Universiti Sultan Zainal Abidin, Kuala Terengganu 20400, Terengganu, Malaysia; 2 Department of Physiology, School of Medical Sciences, Universiti Sains Malaysia, Kubang Kerian 16150, Kelantan, Malaysia; 3 Department of Science Laboratory Technology, Akanu Ibiam Federal Polytechnic, Unwana P.M.B. 1007, Afikpo, Ebonyi State, Nigeria; 4 Institute for Research in Molecular Medicine, Universiti Sains Malaysia, Kubang Kerian 16150, Kelantan, Malaysia; 5 Department of Pathology, School of Medical Sciences, Universiti Sains Malaysia, Kubang Kerian 16150, Kelantan, Malaysia; 6 Unit of Integrative Medicine, School of Medical Sciences, Universiti Sains Malaysia, Kubang Kerian 16150, Kelantan, Malaysia

**Keywords:** orlistat, obesity, oxidative stress, inflammation, apoptosis

## Abstract

Oxidative stress contributes to major complications of obesity. This study intended to identify whether orlistat could mitigate myocardial damage in obese animal models. The tested rats were divided into two groups and fed either with normal chow (*n* = 6 per group) or with a high-fat diet (HFD) for 6 weeks to induce obesity (*n* = 12 per group). Obese rats were further subjected to treatment either with distilled water (OB group) or orlistat 10 mg/kg/day (OB + OR group). Key indices of oxidative stress, inflammation, and apoptosis were assessed using an immunohistochemical-based technique and real-time PCR. The OB group showed significant increases of oxidative stress markers (TBARs and PCO), with significant decreases of anti-oxidant markers (Nrf2, SOD, CAT, and GPx). Furthermore, mRNA expression of pro-inflammatory markers (TNF-α and NF-κβ) and pro-apoptosis markers (Bax, Caspase-3, Caspase-8, and Caspase-9) were significantly upregulated in the OB group. Obese rats developed pathological changes of myocardial damages as evidenced by the presence of myocardial hypertrophy and inflammatory cells infiltration. Orlistat dampened the progression of myocardial damage in obese rats by ameliorating the oxidative stress, and by inhibiting NF-κβ pathway and caspase-dependent cell apoptosis. Our study proposed that orlistat could potentially mitigate oxidative stress-linked myocardial damage by mitigating inflammation and apoptosis, thus rationalizing its medical usage.

## 1. Introduction

In the 21st century, obesity has threatened the health system and the quality of life of millions of people from all walks of life. Generally, obesity is described as an excess accumulation of stored fat in the body. Obesity has implicated numerous diseases that are associated with the complications that arise due to prolonged accumulation of fat in the body. These include cardiovascular diseases (CVD) [1]. Obesity progression greatly impacts the structural and functional status of the heart and is often related to cardiomyopathy [2]. Over the past decades, a number of obesity-induced animal models have been developed. The Sprague Dawley rat is a standard type of rodent used for high-fat diet (HFD)-induced obesity due to its higher susceptibility to obesity development [3].

Oxidative stress could play a major causative role in the development of obesity via the generation of free radicals and reactive oxygen species. The enzymatic antioxidant system works primarily as a first-line defender to suppress free radicals and the imbalance in oxidative stress, which includes superoxide dismutase (SOD), glutathione peroxidase (GPx), and catalase (CAT) [4]. To maintain cell integrity and prevent cell damage, the cells are well-coordinated following activation of nuclear factor erythroid 2-related factor-2 (Nrf2), which is crucial for initiating the generation of antioxidant enzymes such as SOD, GPx, CAT, glutathione S-transferase (GST), heme oxygenase 1, and NAD(P)H none oxidoreductase by binding to the antioxidant response element in the promoter of genes [5].

It is generally considered that inflammation is an important pathogenic factor for obesity progression and complication. The nuclear factor kappa β (NF-κβ) is a family of transcription factors involved in the regulation of inflammatory response, where it initiates the expression of inflammatory response target genes, including tumour necrosis factor α (TNF-α) [6]. Experimental animals using hyperlipidaemic C57/BL6 mice demonstrated the presence of cardiac inflammation following 16 weeks of HFD administration. Significant upregulations in myocardial mRNA expressions of pro-inflammatory cytokines, namely *TNF-α*, and *interleukin (IL)-6*, were also associated with abnormal histological findings of myocardial tissue such as cardiac hypertrophy and fibrosis [7]. Oxidative stress and inflammation are not only correlated, but their imbalance can lead to cell apoptosis and severe complications from obesity. Data from experimental studies have indicated two major pathways in regulating cardiomyocyte and aortic cell apoptosis in obese animals. The pathways involved are extrinsic Fas receptor-dependent (type 1) and intrinsic mitochondria-dependent (type 2) [8]. 

Orlistat is the most widely available prescribed pharmaceutical drug for reducing body fat mass. Additionally, it has also concomitantly caused positive effect on the reduction of cardiometabolic risk factors such as blood pressure, lipid profiles, atherogenic index, insulin, and Homeostatic Model Assessment for Insulin Resistance (HOMA-IR) levels in obese patients treated with orlistat [9,10]. Recent data on pharmacological studies have shown that orlistat works by dose-dependent effect in fecal loss by inhibiting the breakdown and absorption of dietary fat. This potent inhibitor of gastropancreatic lipase works selectively and causes a maximum inhibition of 30% of fat from dietary meals. Among all anti-obesity drugs, orlistat is the most preferable due to its performance in causing the fewest gastrointestinal side effects such as oily spotting, flatulence, fecal incontinence, steatorrhea, and fecal urgency [11,12,13].

However, to date, it is not known whether orlistat may also have therapeutic effect on obesity-related cardiac complications such as cardiac hypertrophy and cardiomyopathy in obese animal models. Therefore, the present study aims to explore the effect of orlistat on myocardium in obese rats, with an emphasis on the pathogenic mechanism related to oxidative stress, inflammation, and apoptosis responses.

## 2. Results

### 2.1. Orlistat Decreased Anthropometrical Measurement in Obese Rats

After 12 weeks of HFD administration, data showed that final weight, weight changes, Lee obesity index, and body mass index (BMI) were significantly increased in the OB group compared to the Normal group. Orlistat treatment was able to significantly attenuate all these obesity parameters compared to the OB group (Table 1).

### 2.2. Orlistat Decreased Lipid Components and Cardiac Enzymes in Obese Rats

The OB group exhibited significant increases of the levels of total cholesterol (TC) and triglyceride (TG) in the serum and cardiac tissue compared to the Normal group. Conversely, obese rats treated with orlistat significantly restored these lipid components in the serum and cardiac tissue. The OB group showed significant increases of the serum levels of creatinine kinase for muscle and brain (CKMB) and lactate dehydrogenase (LDH) compared to the Normal group. However, the orlistat-treated group ameliorated the release of these enzymes into the serum (Table 2).

### 2.3. Orlistat Reduced Cardiac Oxidative Stress Markers in Obese Rats

The OB group showed significant increases of the levels of thiobarbituric acid reactive substances (TBARS) and protein carbonyl (PCO) in the heart, along with significant decreases of the activities of SOD, CAT, and GPx system (GPx, GR, and GST) compared to the Normal group. However, the orlistat-treated group exhibited significant decreases of cardiac TBARS and PCO levels, as well as significant increases of cardiac SOD, GR, and GST activities compared to the the OB group (Table 3).

### 2.4. Orlistat Increased mRNA Levels of Cardiac Antioxidant Enzymes in Obese Rats

**Figure 1:**
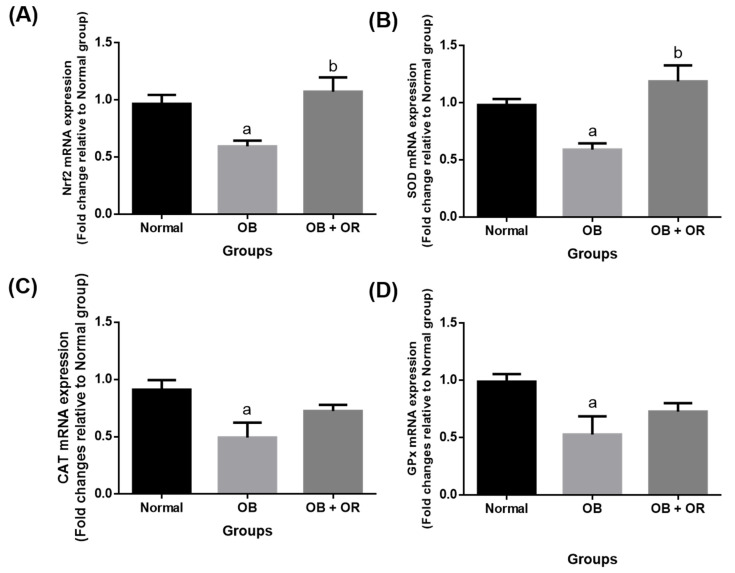
Cardiac mRNA expressions of (**A**) *Nrf2*, (**B**) *SOD*, (**C**) *CAT*, and (**D**) *GPx* in Normal, OB (Obese control), and OB + OR (obese and orlistat 10 mg/kg/day) groups. Values are presented in mean and SEM, *n* = 6 rats per group. ^a^ *p* < 0.05 compared with Normal group, ^b^ *p* < 0.05 compared with OB group. Data were analyzed by One-way ANOVA followed by Tukey’s post-hoc test. CAT; catalase, GPx; glutathione peroxidase, Nrf2; nuclear factor erythroid factor-2, SOD; superoxide dismutase.

The OB group showed a significant downregulation in mRNA expressions of *Nrf2*, *SOD, CAT*, and *GPx* in the heart of obese rats when compared to the Normal group. Meanwhile, the administration of orlistat to obese rats significantly upregulated the mRNA expressions of *Nrf2* and *SOD* compared to the OB group (Figure 1).

### 2.5. Orlistat Improved mRNA and Protein Levels of Cardiac Inflammation Markers in Obese Rats

**Figure 2:**
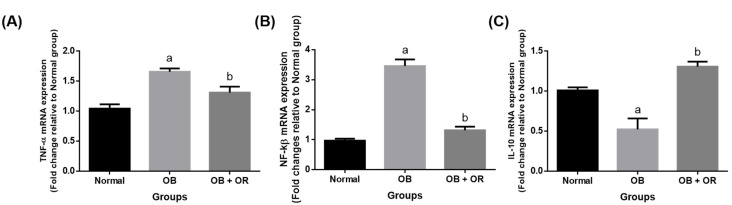
Cardiac mRNA expressions of (**A**) *TNF-α*, (**B**) *NF-ĸβ*, and (**C**) *IL-10* in Normal, OB (Obese control), and OB + OR (obese and orlistat 10 mg/kg/day) groups. Values are presented in mean and SEM, *n* = 6 rats per group. ^a^ *p* < 0.05 compared with Normal group, ^b^ *p* < 0.05 compared with OB group. Data were analyzed by One-way ANOVA followed by Tukey’s post-hoc test. TNF-α; tumour necrosis factor-α, NF-ĸβ; NF-κβ: nuclear factor kappa β, IL; interleukin.

The mRNA expressions of the pro-inflammatory markers of *NF-κβ* and TNF-α were significantly increased in the OB group compared to those rats in the Normal group. Furthermore, the mRNA expression of the anti-inflammatory marker of *IL-10* was significantly decreased in the OB group compared to the Normal group. The imbalance in pro- and anti-inflammatory markers were reverted in the orlistat-treated group, as evidenced by significant decreases of *NF-κβ* and *TNF-α* genes, with a significant increase of the *IL-10* gene observed in the OB + OR group compared to the OB group (Figure 2).

**Figure 3:**
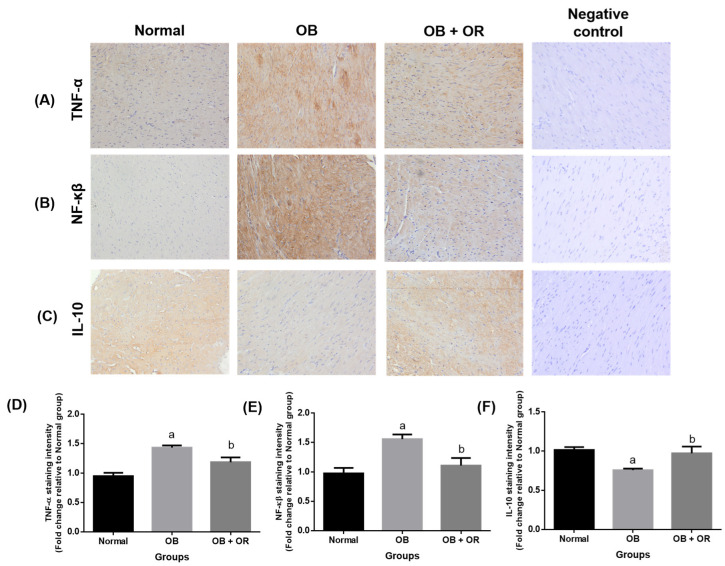
Effects of orlistat on immunoexpressions of (**A**) TNF-α, (**B**) NF-kβ, and (**C**) IL-10 in cardiac section of rats in Normal, OB (obese control), OB + OR (obese and orlistat 10 mg/kg/day) and negative control groups (magnification = ×400, scale bar = 50 μm). For each target protein, phosphate buffered saline was substituted for the primary antibody in the negative control experiment. Staining intensities are shown in (**D**–**F**) and presented as mean and S.E.M values for respective proteins; *n* = 6 rats per group. ^a^ *p* < 0.05 compared with Normal group, ^b^ *p* < 0.05 compared with OB group. Data were analyzed by One-way ANOVA followed by Tukey’s post-hoc test.

Immunohistochemical findings demonstrated significant increases of the expressions of NF-κβ and TNF-α in association with a significant decrease of IL-10 expression in the cardiac tissue of the OB group compared to the Normal group. Meanwhile, orlistat treatment significantly decreased the levels of NF-κβ and TNF-α pro-inflammatory markers in association with a significant increase of IL-10 protein immunoexpression in the OB + OR group compared to the OB group (Figure 3).

### 2.6. Orlistat Improved mRNA and Protein Levels of Cardiac Apoptotic Markers in Obese Rats

**Figure 4:**
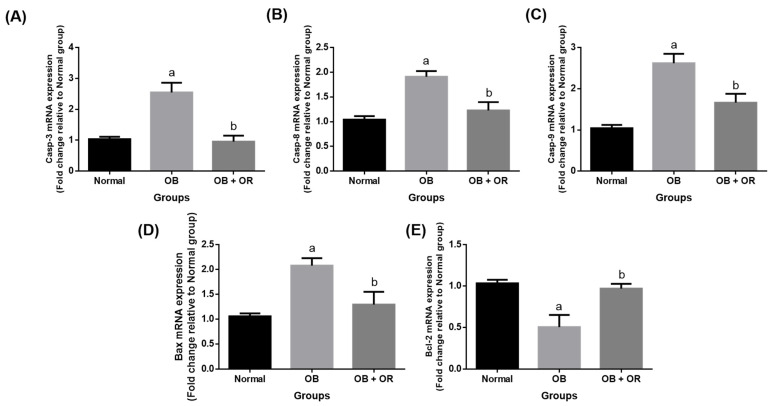
Cardiac mRNA expression of (**A**) *Casp-3*, (**B**) *Casp-8*, (**C**) *Casp-9*, (**D**) *Bax*, and (**E**) *Bcl-2* in Normal, OB (Obese control), and OB + OR (obese and orlistat 10 mg/kg/day) groups. Values are presented in mean and S.E.M, *n* = 6 rats per group. ^a^ *p* < 0.05 compared with Normal group, ^b^ *p* < 0.05 compared with OB group. Data were analyzed by a One-way ANOVA followed by Tukey’s post-hoc test. Bax; Bcl-2 associated X protein, Bcl-2; β-cell lymphoma-2, Casp; caspase.

The OB group showed significant increases of the myocardial mRNA expressions of *Bcl-2 associated X protein* (*Bax*), *caspase* (*Casp*)*-8*, *Casp-9*, and *Casp-3* along with a significant decrease of the mRNA expression of *β-cell lymphoma-2* (*Bcl-2*) compared to the Normal group. Additionally, administration of orlistat to the obese rats significantly decreased the mRNA expressions of *Bax*, *Casp-8*, *Casp-9*, and *Casp-3* while significantly increasing the mRNA expression of *Bcl-2* (Figure 4).

**Figure 5:**
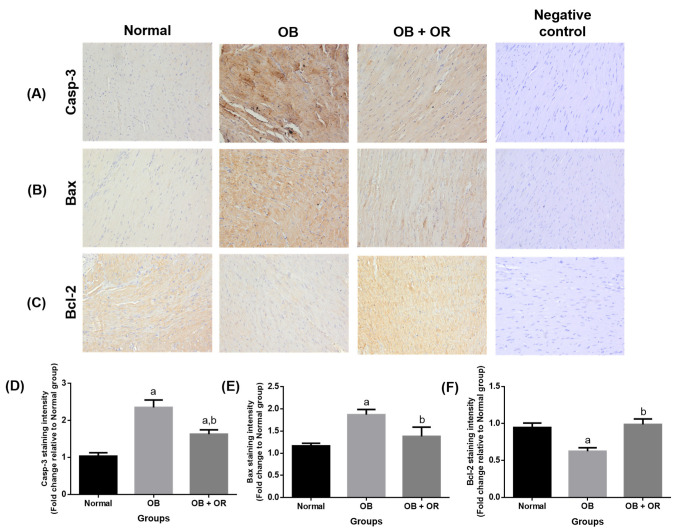
Effects of orlistat on immunoexpression of (**A**) Casp-3, (**B**) Bax, and (**C**) Bcl-2 in cardiac section of rats in Normal, OB (obese control), OB + OR (obese and orlistat 10 mg/kg/day) and negative control groups (magnification = ×400, scale bar = 50 μm). For each target protein, phosphate buffered saline was substituted for the primary antibody in the negative control experiment. Staining intensities are shown in (**D**–**F**) and presented as mean and SEM values for respective proteins; *n* = 6 rats per group. ^a^ *p* < 0.05 compared with Normal group, ^b^ *p* < 0.05 compared with OB group. Data were analyzed by One-way ANOVA followed by Tukey’s post-hoc test. Bax; Bcl-2 associated X protein, Bcl-2; β-cell lymphoma-2, Casp; caspase.

The OB group revealed significant increases of the immunoexpression of Casp-3 and Bax proteins, with a significant decrease of the immunoexpression of Bcl-2 protein compared to the Normal group. The imbalance in pro-and anti-apoptotic proteins was significantly reverted as significant decreases of Casp-3 and Bax immunoexpressions were observed in the OB + OR group, along with a significant increase of Bcl-2 immunoexpression compared to the OB group (Figure 5).

### 2.7. Effect of Orlistat on Cardiac Histology

**Figure 6:**
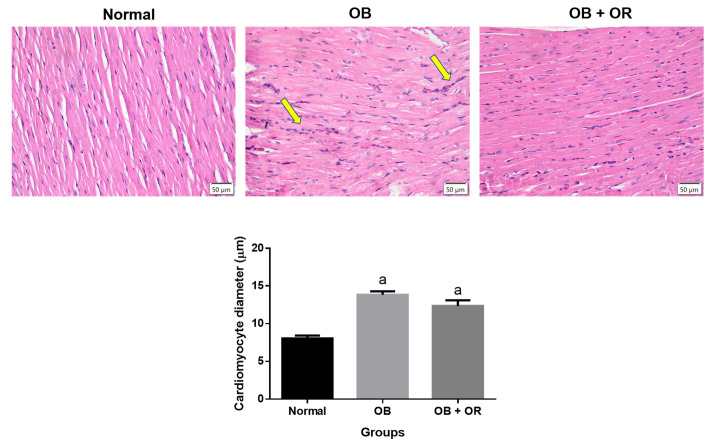
Representative images of H&E-stained left ventricle in longitudinal sections in Normal, OB (Obese control), and OB + OR (obese and orlistat 10 mg/kg/day) groups (magnification ×400, scale bar = 50 μm). Normal group revealed a normal architecture of cardiomyocyte cells which were lined in homogenous pattern. The OB and OB + OR groups exhibited significant increases of cardiomyocyte diameter when compared to Normal group in conjunction with high infiltration of inflammatory cells in OB group (yellow arrow). ^a^ *p* < 0.05 compared with Normal group. Data were analyzed by One-way ANOVA followed by Tukey’s post-hoc test.

A cardiomyocyte longitudinal section was used to measure cardiomyocyte diameter using H&E staining. Microscopic examination of heart tissue in the Normal group revealed a normal architecture of cardiomyocyte cells, which were lined in a homogenous pattern. The OB and OB + OR groups exhibited a significantly larger size of cardiomyocyte cells compared to the Normal group in conjunction with higher infiltration of inflammatory cells in the OB group. Although it is not statistically significant, orlistat showed a tendency to ameliorate cardiomyocyte hypertrophy, as indicated by the slightly lower diameter range of cardiomyocyte compared to the OB group (Figure 6).

## 3. Discussion

Obesity has become a strong independent risk factor that accounts for various CVDs [14]. HFD was widely employed to explore the pathophysiology of obesity and its related complications in view of its characteristics that closely mimic human obesity. Scientific evidence has demonstrated that orlistat exhibits a wide range of pharmacological activities, for example, anti-obesity [9], anti-inflammatory [15], and anti-tumour [16] activities, as well as improved oxidative stress status in the brain, renal, testis, and liver organs [17,18,19]. In our earlier studies, orlistat that was given to HFD-fed rats for 6 and 12 weeks, had shown to significantly improve the oxidant–antioxidant status in the heart and aorta tissues [11,13]. The results of the present study demonstrated that orlistat that was given for 6 weeks after the induction of obesity to an obese rat model, had the potential to reduce myocardial damage in obese rats by mitigating oxidative stress, inflammation, and apoptosis pathways.

The administration of HFD for 12 weeks resulted in excessive increases of obesity parameters, as indicated by the significant increases of body weight gain, Lee obesity index, and BMI in the OB group compared to the Normal group. Furthermore, continuous HFD administration for 12 weeks also caused significant increases of serum TC and TG levels in the OB group compared to the Normal group, suggesting that by using an obese rat model which resulted from HFD administration, we are able to show that obesity is associated with high body weight and hyperlipidaemia. Orlistat treatments for 6 weeks to obese rats have shown to successfully deviate all these abnormalities. This might indicate that orlistat exhibits anti-obesity and anti-hyperlipidaemic activity, which is in line with other studies [19,20]. This could be due to its potent effect in inhibiting gastrointestinal lipase, thus preventing the absorption of dietary lipids at the intestinal level [21].

High dietary fat intake has led to an increase of the circulating fatty acids that are further stored in the form of triacylglycerol in the tissues and fat cells [22]. The increased circulating lipids can also deposit in between the cell tissues and cause damage to the cell homeostasis system [23]. This is consistent with our finding on the increased levels of TC and TG found in the cardiac tissue of the OB group. One previous study indicated that higher cardiac lipid content was related to the exhaustion of mitochondrial fatty acid oxidation, which could increase the risk for cardiac incompetence [24]. This could be due to its role as a fatty acid transporter in the heart that can migrate fatty acids into the heart such as fatty acid binding protein, fatty acid translocase, and fatty acid transport proteins 1 and 6 [25,26]. Apart from reducing the TC and TG levels in the serum, orlistat administration for 6 weeks to obese rats seems able to prevent accumulation of these lipid contents in the heart. It was reported that HFD feeding could induce hyperlipidaemia and promote myocardial damage and dysfunction in experimental rats [27]. As indicated in the present study, 12 weeks of administration of HFD had resulted in significant increases of CKMB and LDH levels in the OB group, which may potentially indicate the present of myocardial damage. However, orlistat significantly restored the levels of these enzymes to near normal level, indicating its ability to reduce myocardial damage, hence mitigating the leakage of intracellular components into systemic circulation.

Lipid is one of precursors for oxidative stress and is commonly credited with a crucial role in obesity-related cardiovascular complications [28]. Free radicals generated within a cell can react with the lipid layer that constructs the cell membrane, thus increasing the production of lipid peroxides, which are measured as TBARs. The oxidized lipids have shown to increase the generation of free radicals, which could damage the endogenous antioxidant enzymes and dampen their production, thereby reducing their scavenging potential [29]. This phenomenon is observed in the hearts of the OB group, which implicates the presence of oxidative stress. Moreover, the significant decrease of SOD activity could be due to its destruction by free radicals, leading to a lack of capacity to neutralize them. Orlistat treatment significantly improved the imbalance in pro- and anti-oxidant markers, which could indicate its protective role against the increased production of free radicals, as evidenced by significant decreases of TBARs and PCO levels compared to the OB group. Furthermore, orlistat was able to restore some of the enzymatic antioxidant activities (SOD, GR, and GST). This could also be due to the role of Nrf2, a redox regulator, that can generate their dependent genes, as evidenced by significant upregulation in the mRNA expressions of *Nrf2* and *SOD* genes in the OB + OR group.

Inflammation has been hypothesized to be a major mechanism for developing obesity-related cardiovascular complications [30]. It is centrally regulated by NF-κβ and its activation further causes increases of downstream-targeted genes of cytokines, chemokines, adhesion molecules, stress response genes, and regulators for apoptosis. Furthermore, activated NF-κβ can trigger the transcription of TNF-α, which is ultimately known to play a part in the pathogenesis of acute and chronic inflammation [6]. The present study demonstrated significant increases of mRNA expressions of *NF-κβ* and *TNF-α* in the cardiac of the OB group, suggesting the ongoing inflammation followed prolonged HFD administration. This is further supported by the significant increases of the immunoexpressions of NF-κβ and TNF-α proteins in the cardiac tissue of the OB group compared to the Normal group. Prolonged NF-kB activation has been reported to play a contributing role in the pathogenic process of cardiac hypertrophy, as indicated by histological finding of cardiac tissue in the OB group [2]. This hypertrophy could also be due to a compensatory mechanism secondary to the increased ventricular pressure in response to the increase of central blood volume and cardiac output [31]. Moreover, levels of mRNA and protein expressions of *IL-10* in cardiac tissue were also lower in the OB group compared to the Normal group. The treatment of obese rats with orlistat significantly reverted the imbalance in pro- and anti-inflammatory markers, suggesting that orlistat exerted an anti-inflammatory effect. The increased IL-10 expression in the OB + OR group might be responsible for the decreased TNF-α expression, as IL-10 can inhibit its generation [32]. This is supported by our finding on a remarkable decrease of the infiltration of inflammatory cells in between cardiomyocytes of the OB + OR group, compared to the OB group. However, orlistat treatment to obese rats had shown to be ineffective in reducing cardiomyocyte hypertrophy.

Previous data has shown that an increase of oxidative stress can activate Bax and disrupt the mitochondria function, thereby activating the caspase pro-apoptotic system [33]. This is supported by a few studies which demonstrated significantly higher apoptotic cell death and damage in obese rats and mice models with involvement of Fas-dependent and mitochondrial-dependent apoptotic pathways [2,34]. In the present study, some markers from the mitochondrial- and Fas-dependent apoptotic system were evaluated. It is demonstrated that both apoptotic pathways are activated in obese condition, as indicated by significant increases of *Bax, Casp-8, Casp-9,* and *Casp-3* mRNA expressions found in the OB group. These findings are also in concomitant with findings on immunohistochemical staining, as evidenced by significant increases of Bax and Casp-3 proteins. The results are further substantiated by significant increases of cardiac enzymes (CKMB and LDH) in the OB group compared to the Normal group. Additionally, *Bcl-2* mRNA expression was significantly decreased in the OB group compared to the Normal group, which could possibly be due to the increased oxidative stress. Treatment of obese rats with orlistat showed significant improvement in the mRNA and protein expressions of these pro- and anti-apoptotic markers compared to the OB group. This could be due to its ameliorative effect against oxidative stress, which leads to reduced apoptotic cell death events.

Our findings suggest that orlistat possesses a beneficial effect in improving obesity-related myocardial damage, potentially by attenuating the level of cardiac lipid content, thereby reducing the oxidative stress, inflammation, and apoptotic changes. This beneficial effect could be also directly related to its effect on reducing body weight in obese rats. Hence, orlistat, which acts by inhibiting gastrointestinal lipase, may be considered as a valuable therapeutic agent in treating myocardial damage in obesity. In future studies, it is recommended to evaluate heart contractility to assess cardiac performance in obese animal models.

## 4. Materials and Methods

### 4.1. Chemicals and Reagents

Formaldehyde solution was purchased from Merck, Darmstadt, Germany. Harris haematoxylin and eosin Y solution were purchased from Sigma Aldrich, St. Louis, MO, USA. Innu Prep RNA mini kit was commercially bought from Analytik Jena, Jena, Germany. Rabbit polyclonal antibodies against Bax, bcl-2, casp-3, IL-10, NF-kB, and TNF-α were purchased from Cloud-Clone Corp, Katy, TX, USA. Dako En VisionTM+ System/HRP. Rb and Dako TMB Substrate for signal detection were generated by Agilent Technologies, Inc., Santa Clara, CA, USA. Primers were procured from Integrated DNA Technologies (IDT, Bayan Lepas, Malaysia). All other reagents were of analytical grade.

### 4.2. Orlistat Preparation

Orlistat was purchased commercially from Xepa-Soul Pattinson Sdn. Bhd. (Melaka, Malaysia) and stored at room temperature. Orlistat powder was weighed according to the rat’s weight and dissolved in 1 mL distilled water before being administered to the rats orally.

### 4.3. Animals and Diet

Animal experiments were carried out in strict accordance with the recommendations in the Guide for the Care and Use of National Institute of Health Guide for the Care and Use of Laboratory Animals and the protocol was approved by the Institutional Animal Care and Use Committee (IACUC), USM [USM/IACUC/2018/(113)(933)]. Male Sprague Dawley rats at 8–10 weeks and of a weight between 180–230 g, which were procured from the Animal Research and Service Centre (ARASC), Universiti Sains Malaysia (USM), were included in the study. These rats were housed in a climate controlled, light-regulated facility with a 12:12 h light/dark cycle. Normal pellet was manufactured from Altromin Spezialfutter GmbH & Co. KG, Lage, Germany. High-fat diet (HFD) was prepared weekly and consisted of 64% powdered normal pellet, 32% animal ghee, and added with 300 mg Calcium and 100 UI Vitamin D3. The ingredients were well mixed and added with 12% cholesterol powder [11]. The HFD mixture was shaped into small hand ball and kept in the -4′C fridge before being fed to the rats the next day.

### 4.4. Animal Experimental Design

The animals were randomly divided into two experimental groups with different types of diet approaches i.e., normal diet (Control group, *n* = 6) and high-fat diet (HFD group, *n* = 12) for 6 weeks to induce obesity. Obese rats in the HFD group were further divided into two groups and received the following treatment for another 6 weeks via oral gavage feeding: (1) OB (distilled water), (2) OB + OR (orlistat 10 mg/kg/day). On the last day of the experimental period, the rats were anaesthetized with 90 mg/kg ketamine and 5 mg/kg xylazine intraperitoneally.

### 4.5. Anthropometrical Measurement

The body weights were measured once per week and on the day of sacrifice. At the end of the experimental study, the following anthropometrical measurements were performed while the rats were under full anaesthesia. The cut-off value for the Lee obesity index was 315 [35], while for body mass index, the value was 0.68 g cm^−2^ [36].

Lee obesity index = _3_√ body weight (g)/naso-anal length (cm) × 1000

Body mass index = body weight (g)/length^2^ (cm^2^)

### 4.6. Sample Collection

Blood was collected in a heparin tube and coagulated for 2 h. The serum was separated by centrifugation (Avanti J-HC, Beckman Coulter, Indianapolis, IN, USA) at 3000 rpm for 10 min. The heart tissue was immediately excised and rinsed with cold phosphate buffer saline (PBS). The left heart was weighed (Sartorius AG, Göttingen, Germany), and homogenized (IKA Labortechnik Co., Ltd., Wilmington, NC, USA) with 10% cold PBS (*w/v*) and used for further biochemical analysis.

### 4.7. Determination of Total Cholesterol and Triglyceride

The total cholesterol and triglyceride in serum and heart homogenate were detected using commercial kits purchased from Qayee-Bio, Life Science, Shanghai, China.

### 4.8. Determination of Cardiac Enzymes

Serum LDH and CKMB were determined by commercially available kits (Qayee-Bio, Life Science, Shanghai, China) according to the principle of double-antibody sandwich ELISA.

### 4.9. Determination of Cardiac Oxidant-Antioxidant Statuses

Thiobarbituric acid reactive substances (TBARS), protein carbonyl (PCO), SOD, CAT, GPx, glutathione reductase (GR), and glutathione S-transferase (GST) were determined in the homogenate of the left heart. Briefly the methods from Chatterjee et al., 2000 [37] and Evans et al., 1999 [38] were followed to evaluate TBARs and PCO, respectively. The activities of SOD and CAT were determined using the methods of Beyer and Fridovich (1987) [39] and Goth (1991) [40], respectively. Meanwhile, GPx and GR activities were assessed following the method described by Dogan et al., 1994 [41] and Carlberg, I. and Mannervik, 1985 [42], respectively. The GST level was determined according to Habig et al., 1974 [43]. Total protein levels were evaluated using a BCA protein assay kit (Boster Biological Technology, Pleasanton, CA, USA) and all samples were normalized to the protein level.

### 4.10. Immunohistochemical Analysis

Part of left ventricle tissue was fixed in 10% formaldehyde solution (Merck, Darmstadt, Germany) within 48–72 h followed by dehydration with graded alcohol concentration using an automated tissue processor (Leica, Wetzlar, Germany). The processed tissue was embedded (Thermo Electron Corporation, Waltham, MA, USA) in paraffin block, sectioned (Thermo Electron Corporation, Waltham, MA, USA) at 5 µm thickness, and mounted on a poly-L-lysine-coated slides. The immunohistochemical analysis was performed according to pressure cooker method. The tissue section was warmed at 60 °C for 20 min followed by immersed in xylene and dehydrated in graded alcohol concentration. Antigen retrieval was performed by placing the tissue section in a pressure cooker containing heated Tris-EDTA buffer with 0.05% Tween 20 (pH 9.0) at 120 °C for 3 min. The tissue section was blocked with 3% hydrogen peroxide in PBS saline for 5 min at room temperature. The section was incubated with primary antibodies (NF-κβ, IL-10, Casp-3, Bax, and Bcl-2) as shown in Table 4, at 4 °C overnight for 24 h except for TNF-α, which was performed at room temperature for 1 h. Then the section was incubated with Dako En VisionTM+ System/HRP. Rb (Agilent Technologies, Inc., Santa Clara, CA, USA) for 1 h at room temperature. All washing steps were performed by rinsing with Tris-buffered saline containing 0.05% tween 20 (pH 8.4). The immunoreaction was visualized following DAB incubation (Dako TMB Substrate Agilent Technologies, Inc., Santa Clara, CA, USA). Section was mounted with cytoseal (Thermo Scientific, Waltham, MA, USA) after brief counterstained with Haematoxylin and rehydration. All sections were viewed under microscope (Olympus BX41, Olympus Co., Tokyo, Japan) at 400× magnification. The mean values of staining intensity of the fields were calculated using ImageJ software (Bethesda, MD, USA) in fold changes relative to the changes in the Normal group as control.

### 4.11. Analysis of Gene Expression

#### 4.11.1. RNA Extraction, Purity and Integrity Assessment

During sacrifice, part of the left heart was cleaned of adhesive blood and rinsed with normal saline before being stored in RNAlater (Sigma-Aldrich, St. Louis, MO, USA) until further use. The total RNA was extracted (Analytik Jena, Jena, Germany), which included 15 min incubation at room temperature with DNAse enzyme to remove gDNA. The RNA purity was assessed by Udrop plate reader (Thermo Fisher Scientific, Finland) to obtain the purity and concentration of extracted RNA. Samples with OD_260/280_ of 1.8–2.0 were checked for the presence of double bands (18 s and 28 s) via UV transilluminator (ChemiDoc XRS, Bio-Rad Laboratories, Hercules, CA, USA) by referring to RNA ladder by gel electrophoresis using 1% agarose in 1× LB buffer. Samples with well-defined and distinct bands were employed for reverse transcriptase-quantitative polymerase chain reaction (rt-qPCR) method.

#### 4.11.2. Real-Time Quantitative PCR

The qPCR was carried out on an AriaMx Real-Time PCR system (Agilent Technologist, Santa Clara, CA, USA) and connected to a Stratagene Mx3000p qPCR real-time machine (Agilent Technologist, Santa Clara, CA, USA). The StepOnePlus Real Time PCR system (Applied Biosystems Co., Foster City, CA, USA) was used with a SensiFAST SYBR Lo-Rox One-Step PCR kit (Bioline, UK) according to the manufacturer’s protocol. The reaction mixtures comprised 10 ng of the RNA template, 0.4 µL each for forward and reverse primers (10 µmol), 0.4 µL of RNase inhibitor, 0.2 µL of reverse transcriptase, 10 µL of SYBR green and adequate DepC treated H_2_O to reach a total volume of 20 µL. The setting for qPCR thermoplate system was as follows: denaturation at 95 °C for 10 min, annealing at 60 °C for 2 min, followed by 40 cycles of 95 °C for 10 s, 60 °C for 5 s and 72 °C for 20 s. Each CT value of the targeted gene was normalized to the CT value of glyceraldehyde 3-phosphate dehydrogenase, respectively, and followed to the calculation by the 2^−∆∆Ct^ method. The primer sequences used in this study are shown in Table 5.

### 4.12. Cardiac Histology

Part of the left ventricle tissue was fixed in 10% formaldehyde solution. The fixed tissue was dehydrated in a graded concentration of alcohol solution using an automated processor machine (Leica, Wetzlar, Germany). The tissue was embedded in paraffin block and sectioned at 5 µm thickness using a rotary microtome (Thermo Electron Corporation, Waltham, MA, USA). The tissue section was stained with haematoxylin and eosin to assess the diameter of cardiomyocyte (10 cardiomyocyte per field, 10 fields per heart section) at 400× magnification [44]. The cardiomyocyte diameter was calculated using the hand-tool section available in Image J software (Bethesda, MD, USA).

### 4.13. Data Analysis

Statistical tests were computed using GraphPad Prism 6.0 software (GraphPad Software Inc., La Jolla, CA, USA). All results were checked for normality using Shapiro–Wilk normality test and assessed for homogenous variance. Data with normal distributions were subjected for One-way analysis of variance (ANOVA) test. All analysed data are presented in mean and standard error of mean (SEM) values. A value of *p* < 0.05 was considered as statistically significant.

## 5. Conclusions

Based on our findings, prolonged administration of HFD to Sprague Dawley rats had shown to promote the development of myocardial damage, as demonstrated by significant increases of cardiac lipid content, oxidative stress, pro-inflammatory, and apoptosis markers, leading to derangement in myocardial alignment, infiltration of inflammatory cells, and myocardial hypertrophy, which eventually might cause cardiac complication. The present study indicates that orlistat, an anti-obesity drug, has pharmacotherapeutic significance against obesity-related myocardial damage through its effect in mitigating oxidative stress, inflammation, and apoptosis. In addition, further study is also suggested to evaluate any possible effects of orlistat on these parameters in rats fed with a normal diet and to also evaluate the vital signs and hemodynamic status, as well as liver, renal, and cardiac functions.

## Figures and Tables

**Table 1:** Effect of orlistat on anthropometrical measurement in obese rats.

Anthropometrical Measurements	Normal	OB	OB + OR
Initial weight (g)	216.3 (7.16)	228.5 (6.91)	228.4 (9.05)
Final weight (g)	335.1 (8.85)	466.5 (11.66) ^a^	407.0 (12.63) ^a,b^
Weight changes (g)	106.7 (5.61)	228.6 (13.46) ^a^	176.2 (9.97) ^a,b^
Lee Obesity index	303.3 (1.79)	327.7 (2.50) ^a^	319.2 (1.72) ^a,b^
BMI (g/cm^2^)	0.67 (0.02)	0.86 (0.03) ^a^	0.77 (0.02) ^a,b^

Data were presented as mean and SEM values, *n* = 6 per group. OB: obese; OB + OR: Obese and orlistat 10 mg/kg/day. ^a^ *p* < 0.05 compared to Normal group, ^b^ *p* < 0.05 compared to OB group (One-way ANOVA followed by Tukey’s post-hoc test).

**Table 2:** Effect of orlistat on lipid components and cardiac enzymes in obese rats.

Parameters	Normal	OB	OB + OR
Serum TC (mmol/L)	1.54 (0.06)	2.81 (0.31) ^a^	2.00 (0.09) ^b^
Cardiac TC (ng/mL)	174.8 (13.65)	265.4 (12.23) ^a^	217.7 (8.14) ^b^
Serum TG (mmol/L)	0.49 (0.03)	0.93 (0.03) ^a^	0.70 (0.05) ^a,b^
Cardiac TG (ng/mL)	233.00 (7.49)	313.9 (15.93) ^a^	256.8 (7.99) ^b^
Serum CKMB (ng/mL)	0.47 (0.03)	0.67 (0.06) ^a^	0.52 (0.02) ^b^
Serum LDH (ng/mL)	0.12 (0.00)	0.20 (0.01) ^a^	0.14 (0.00) ^b^

Data were presented as mean and SEM values, *n* = 6 per group. OB: obese; OB + OR: Obese and orlistat 10 mg/kg/day. ^a^ *p* < 0.05 compared to Normal group, ^b^ *p* < 0.05 compared to OB group (One-way ANOVA followed by Tukey’s post-hoc test). CKMB: creatinine kinase for muscle and brain; LDH: lactate dehydrogenase; TC: total cholesterol; TG: triglyceride.

**Table 3:** Effect of orlistat on oxidant-antioxidant statuses in obese rats.

Oxidant-Antioxidant Statuses	Normal	OB	OB + OR
TBARs (nmol/mg protein)	1.74 (0.14)	19.47 (0.56) ^a^	8.03 (1.03) ^a,b^
PCO (nmol/mg protein)	1.02 (0.18)	2.31 (0.31) ^a^	1.18 (0.29) ^b^
SOD (U/mg protein)	100.7 (4.85)	70.29 (2.74) ^a^	119.2 (5.88) ^a,b^
CAT (U/mg protein)	81.05 (6.55)	44.81 (4.13) ^a^	57.79 (3.31) ^a^
GPx (U/mg protein)	1.96 (0.18)	0.74 (0.14) ^a^	1.15 (0.15) ^a^
GR (U/mg protein)	0.70 (0.07)	0.21 (0.05) ^a^	1.00 (0.15) ^b^
GST (U/mg protein)	9.52 (0.71)	6.00 (0.66) ^a^	10.44 (0.98) ^b^

Data were presented as mean and SEM values, *n* = 6 per group. OB: obese; OB + OR: Obese and orlistat 10 mg/kg/day. ^a^ *p* < 0.05 compared to Normal group, ^b^ *p* < 0.05 compared to OB group (One-way ANOVA followed by Tukey’s post-hoc test). CAT: catalase; GPx: glutathione peroxidase; GR: glutathione reductase; GST: glutathione S-transferase; PCO: protein carbonyl; SOD: superoxide dismutase; TBARs: thiobarbituric acid reactive substances.

**Table 4:** Rabbit polyclonal antibodies used in the study.

Primary	Reference Number	Concentration	Incubation Temperature
TNF-α	PAA133Ra01	1:120	RT
NF-κβ	PAB824Ra01	1:100	4 °C
IL-10	PAA056Ra01	1:100	4 °C
Casp-3	PAA626Ra01	1:150	4 °C
Bax	PAB343Ra01	1:100	4 °C
Bcl-2	PAA778Ra01	1:50	4 °C

Bax; Bcl-2 associated X protein; Bcl-2: β-cell lymphoma; Casp; caspase, IL; interleukin, NF-κβ; nuclear factor kappa β, TNF-α; tumour necrosis factor-α.

**Table 5:** List of primer sequences.

Gene	Accession Number	Forward (5′-3′)	Reverse (3′-5′)	Size (bp)
*SOD*	X05634.1	CGAGCATGGGTTCCATGTC	CTGGACCGCCATGTTTCTTAG	101
*CAT*	NM_012520.2	ACAACTCCCAGAAGCCTAAGAATG	GCTTTTCCCTTGGCAGCTATG	76
*GPx*	NM_030826.4	GGAGAATGGCAAGAATGAAGA	CCGCAGGAAGGTAAAGAG	139
*Nrf2*	NM_031789.1	CAGGTTGCCCACATTCCCAA	ATATCCAGGGCAAGCGACTCAT	110
*TNF-α*	NM_012675.3	ACTGAACTTCGGGGTGATCG	GCTTGGTGGTTTGCTACGAC	153
*IL-10*	NM_012854.2	TTGAACCACCCGGCATCTAC	CCAAGGAGTTGCTCCCGTTA	91
*NF-κβ*	NM_199267.2	CGCGGGGACTATGACTTGAA	AGTTCCGGTTTACTCGGCAG	163
*Casp-3*	NM_012922	AAGATACCAGTGGAGGCCGACTTC	GGGAGAAGGACTCAAATTCCGTGG	199
*Casp-8*	NM_022277.1	GTTCTCTCAGTTGCCTTTCTCC	GGCCAGTCCGCCAAAGTTTA	90
*Casp-9*	NM_031632	CTGAGCCAGATGCTGTCCCATA	CCAAGGTCTCGATGTACCAGGAA	168
*Bax*	U49729.1	CGCGTGGTTGCCCTCTTCTACTTT	CAAGCAGCCGCTCACGGAGGA	124
*Bcl-2*	NM_016993.1	ATCGCTCTGTGGATGACTGAGTAC	AGAGACAGCCAGGAGAAATCAAAC	134

Bax; Bcl-2 associated X protein; Bcl-2: β-cell lymphoma; Casp: caspase; CAT: catalase; GPx: glutathione peroxidase; IL-10: interleukin-10; NF-κβ: nuclear factor kappa β; Nrf2: nuclear factor erythroid 2-related factor-2; SOD: superoxide dismutase; TNF-α: tumour necrosis factor alpha.

## Data Availability

Data is contained within the article.
